# A Spatio‐Temporal Diffusion Model for Cardiac Real‐Time Imaging

**DOI:** 10.1002/mrm.70303

**Published:** 2026-02-18

**Authors:** Oliver Schad, Julius Frederik Heidenreich, Nils Petri, Viktor Hartung, Alena Kollmann, Thorsten Alexander Bley, Peter Nordbeck, Bernhard Petritsch, Tobias Wech

**Affiliations:** ^1^ Department of Diagnostic and Interventional Radiology University Hospital Würzburg Würzburg Germany; ^2^ Department of Internal Medicine I University Hospital Würzburg Würzburg Germany; ^3^ Comprehensive Heart Failure Center Würzburg Würzburg Germany

**Keywords:** cardiac imaging, diffusion models, generative modeling, heart, machine learning, magnetic resonance imaging (MRI), video diffusion model

## Abstract

**Purpose:**

Real‐time imaging of cardiac function is favorable due to shorter scan times and becomes necessary when arrhythmia or inability to hold breath leads to insufficient quality of electrocardiogram (ECG)‐gated Cartesian cine. However, comparable spatio‐temporal resolution can only be achieved in undersampled settings, which in turn demand performant reconstruction methods. This study investigates image quality improvements using a novel spatio‐temporal diffusion‐based reconstruction, applied to accelerated spiral real‐time acquisitions.

**Methods:**

In a clinical study, real‐time acquisition was performed using accelerated spiral sampling patterns acquired during breath hold and free‐breathing. Retrospective binning enabled calculation of segmented spiral cine images, which were used to train a spatio‐temporal diffusion model. Reconstruction of accelerated acquisitions was performed using the proposed model, as well as a 2D spatial diffusion model and compressed sensing‐based techniques for comparison. Reconstruction quality was assessed by calculating quantitative image metrics for breath‐held data and by means of an expert‐reader study for free‐breathing scans.

**Results:**

Real‐time acquisitions enabled significantly shorter scan durations in comparison to clinical cine, with improved quality for participants with irregular heartbeats. Quantitative image metrics indicate superior image quality of the proposed method compared to the baseline methods. Expert reader scores imply consistent sharpness and reduced apparent noise for the proposed model.

**Conclusion:**

Including temporal information within the diffusion model improved consistency between frames, reduced noise, and preserved sharpness in the reconstructions of undersampled spiral acquisitions. Long reconstruction times and demanding computational burdens are obstacles to overcome.

## 1 Introduction

To date, electrocardiogram‐gated (ECG) cardiac cine magnetic resonance imaging (MRI) with Cartesian readouts across several RR cycles remains the standard approach to assess cardiac function in clinical routine. However, repeated breath‐held acquisitions lead to long overall scan durations and can cause motion artifacts or patient discomfort. Moreover, the exam can fail in patients with severe arrhythmia due to incorrect gating [1, 2, 3].

A variety of techniques have been proposed for cardiac real‐time imaging, which can largely overcome these limitations. Early approaches relied on Cartesian sampling and parallel imaging (PI) [4, 5]; however, temporal and/or spatial resolution still had to be sacrificed with respect to gated acquisitions. Compressed sensing (CS) and low rank techniques have then shown potential to push acceleration factors beyond unregularized PI in the last decade [6, 7].

Rapid advancements in the field of deep learning (DL) have more recently demonstrated the capability to surpass traditional hand‐crafted approaches. Especially, unrolled gradient schemes provide a natural and powerful data‐driven extension to wavelet‐ or total variation‐based CS [8, 9]. Related studies include further developments of variational networks [10, 11], such as the CineVN [12], which combines a 2D‐t network architecture with data‐consistency operations for low‐latency accelerated cine reconstruction. Others focus on training without paired reference data, as the latter can only be acquired in cardiac MRI by assumptions of periodicity in the heartbeat. Here, various deep image prior approaches have shown promising potential in the field of cardiac real‐time MRI [13, 14, 15, 16]. Similarly, neural implicit representations aim to model continuous signals by mapping coordinate values, thereby avoiding non‐uniform Fourier transforms in the case of non‐Cartesian sampling and enabling flexible temporal interpolation of cardiac cine [17, 18, 19].

In recent studies, generative diffusion models have emerged as a class of neural networks with outstanding modeling opportunities [20, 21, 22, 23, 24, 25] and have thus also been proposed for various MRI reconstruction tasks. As these networks often rely on demanding computational resources, approaches were first restricted to 2D applications [26, 27, 28]. However, more recently, also 3D or 2D + t applications were investigated by providing new conceptual approaches to reduce computational demands, such as direct application in a latent space [29], patch‐based processing [30] or iterative 2D processing with additional 3D regularization [31, 32, 33].

In this paper, we propose a spatio‐temporal diffusion model for the reconstruction of accelerated, spiral cardiac real‐time acquisitions, and compare it to a 2D spatial diffusion model as well as to CS‐baseline methods. Unlike previously published work [27], which individually processes single frames by a 2D diffusion model, this novel approach improves temporal consistency by leveraging spatio‐temporal modeling on a GPU with 48GB of VRAM.

## 2 Methods

### 2.1 MR Reconstruction Problem

The MR reconstruction problem for dynamic images $x\in {\mathbb{C}}^{N_{\mathrm{fr}}\times n\times m}$ can be formulated through the following forward model:

y=MℱSx+ϵ,

where $N_{\mathrm{fr}}$ corresponds to the number of successive images to be reconstructed with the size of spatial dimensions n×m. Generally, time‐resolved raw k‐space data $y\in {\mathbb{C}}^{N_{\mathrm{fr}}\times N_{c}\times N_{S}}$ consist of $N_{S}$ acquired samples per frame, which are measured using multiple receiver coils with number $N_{c}$. The operator S models the weighting of image data with coil‐sensitivities. A spatial Fourier transform ℱ creates k‐space data, which are sampled at discrete locations, as defined by the sampling mask M. In the case of non‐Cartesian sampling, k‐space data can be preprocessed and transferred onto Cartesian grids, for example, by GRAPPA Operator Gridding (GROG) [34] in combination with standard Fourier transformations, or by using a non‐uniform Fourier transform, which maps an image on a Cartesian grid to the non‐Cartesian sampled k‐space locations and vice versa. Altogether, the measurement operator A can be expressed as A=MℱS. In the forward model, *ϵ* accounts for measurement noise. Inverting this equation results in an optimization problem

$$x^{*}=\underset{x}{\arg\;\min}\;{\left|\left|\mathrm{Ax}-y\right|\right|}_{2}^{2}+\lambda \psi \left(x\right),$$

which can additionally be regularized with assumptions of prior knowledge in the acquired data by using a general regularizing function ψ(x) with weighting λ. In the case of diffusion models, a prior over the image space is introduced by a network trained to sample from the distribution of realistic MR images. This prior can then be combined with a likelihood term derived from the MRI measurement model to enable data‐consistent image reconstruction [24].

### 2.2 Data Acquisition for Cardiac Real‐Time Imaging and Volunteer Study

For detailed information on the acquisition procedure, we also refer to previous works using the same dataset [27, 35].

Accelerated cardiac real‐time imaging was performed using a spiral bSSFP sampling scheme (see Figure S1). Thirteen equidistant spiral arms were combined to form undersampled data for a single real‐time frame (temporal footprint of ˜48 ms). A dedicated rotation scheme was used across multiple frames that additionally enabled the reconstruction of segmented spiral cine for subjects with regular heart rates in breath‐held acquisitions. These “spiral segmented cine” consisted of a total of 104 equidistant spiral arms for each cardiac phase, accumulated from ˜8 consecutive heart beats using retrospective DC‐gating based on the central k‐space point.

To avoid time consuming (de)‐gridding operations of the spiral data during the iterative reconstruction schemes, all spiral acquisitions were transferred onto 512px × 512px Cartesian grids using GROG [34]. GRAPPA Kernels were calculated from temporally averaged convolution gridded estimates. Single coil images were combined based on coil‐sensitivity maps, which were estimated from data averaged across all timeframes using ESPIRiT [36] in BART [37]. Here too, GROG was used for a prior transfer onto a Cartesian grid. As no coil‐compression was performed, acquisitions contained 24–34 single coil images.

In a study approved by the local ethics committee under license ID 173/22_skpm, a total of 16 healthy participants and 5 patients with atrial fibrillation were examined on a 1.5 T clinical whole‐body scanner (Magnetom Avanto, Siemens Healthineers, Germany). Data were acquired in left‐ventricular short axis (SAX) slice orientations from base to apex, in free‐breathing as well as breath‐hold. Data from 8 healthy participants were used for training of the proposed model; the remaining data (8 healthy participants, 5 arrhythmic patients) were used for performance evaluation.

### 2.3 Score‐Based Video Diffusion Model

#### 2.3.1 Model Architecture and Training

To model the underlying data distribution of dynamic cardiac MR‐data, we incorporated a spatio‐temporal 2D + t‐Unet architecture [38]—termed “video diffusion model” and based on the original work by Ho et al. [39]—in a score‐based diffusion framework [22].

To not exceed hardware limitations (i.e., GPU memory), the following approaches were employed:

Firstly, the model was trained on (complex) coil‐combined images. Secondly, gradient checkpointing was exploited, trading available GPU memory for prolonged training times. Thirdly, the video diffusion network was trained with a fixed matrix size of 18 frames × 304px × 304px. However, as the network is based on a convolutional network architecture with a variable‐length temporal attention mechanism, processing of variable input dimensions is possible.

Training data consisted of 96 in‐house acquired, segmented spiral cine series from the aforementioned 8 healthy participants. Since the number of spiral cine frames ranged between $N_{\mathrm{fr}}=20-25$ and acquisitions were not started by a trigger, a random circular shift with respect to the succession of cardiac phases was introduced during training. To match the input dimensions of the network during training, a central crop was applied and frames $N_{\mathrm{fr}}>18$ were discarded.

We trained the diffusion model on a NVIDIA RTX A6000 (48 GB memory) with complex‐valued images as two‐channel input. Images were normalized with respect to the maximum value of the respective magnitude images.

#### 2.3.2 Diffusion Posterior Sampling

**FIGURE 1:**
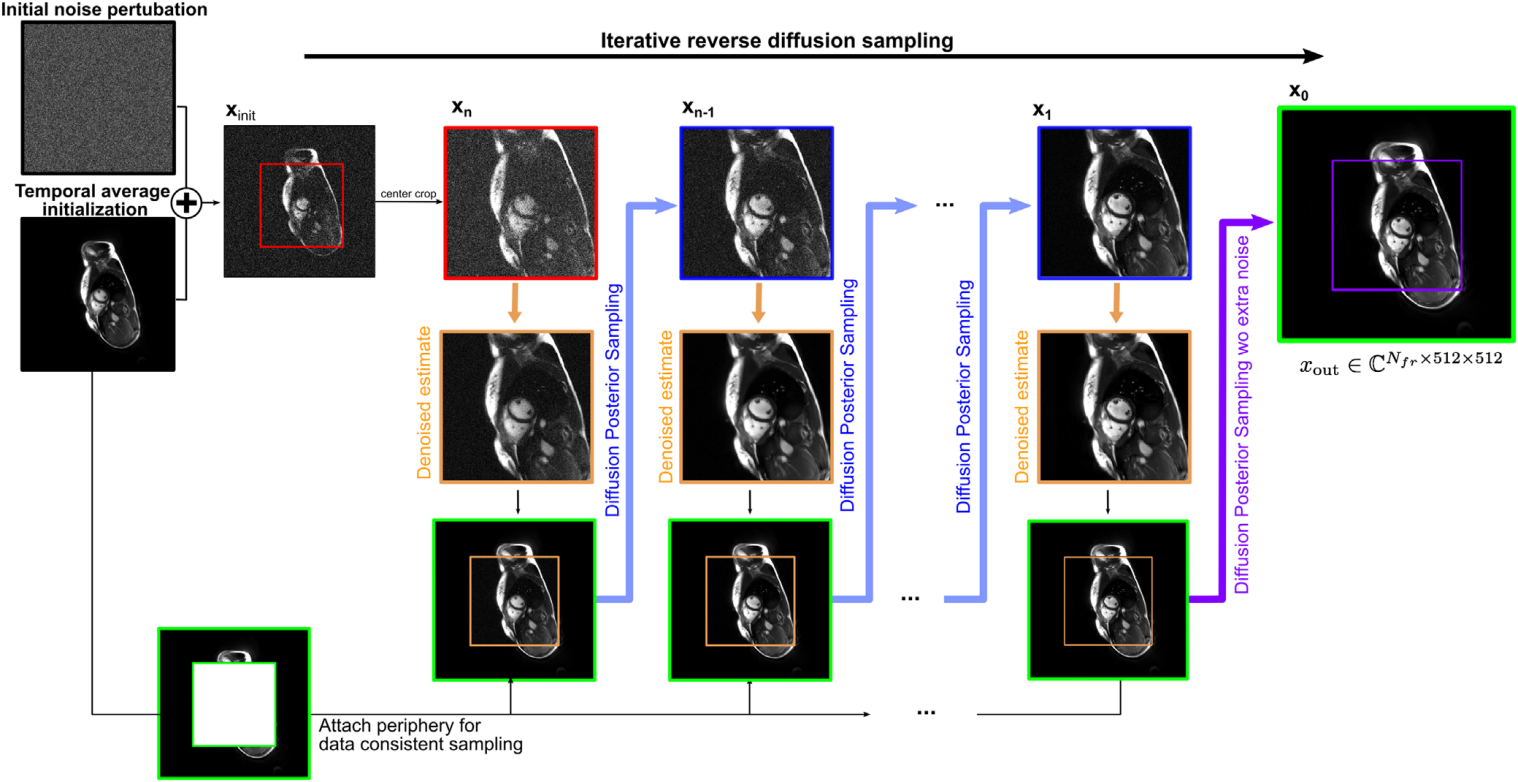
Schematic overview of the proposed reconstruction procedure using a video diffusion model. Single images represent a full temporal time series consisting of $N_{\mathrm{fr}}$ frames for an uncluttered depiction.

The trained diffusion model was then used to reconstruct undersampled spiral real‐time acquisitions (see Figure 1). Inspired by the works of Chung et al. [26, 40], we initialized the reverse sampling procedure with a noise‐perturbed reconstruction estimate and only ran the diffusion chain for a reduced number of steps. To do so, we used the temporal average reconstruction as initialization of the diffusion chain for each temporal frame. To maintain our memory restrictions, the actual model worked only on a central part of the initially gridded reconstruction (304px × 304px), rather than the full field‐of‐view (512px × 512px). We therefore assume that the periphery in image space can be reliably represented by the temporal average and only the central part requires a temporally varying depiction. FOV relations shown in Figure 1 are typical, such that the periphery contains only a small part of the anatomy and mainly encodes background. Since memory constraints represent the primary motivation for this approach, we report estimated memory requirements for the reconstruction of full‐resolution cine data in the Supporting Information as well as Figure S2.

By applying the unconditionally trained score network within the reverse diffusion sampler proposed by Song et al. [22], iterative data generation from the trained probability distribution can be achieved. Diffusion Posterior Sampling (DPS) [41] was used to perform data consistency. DPS requires tracking of gradients also during inference, so that gradient checkpointing was also employed here. As the diffusion chain starts with a static initialization, DPS must enforce cardiac motion in agreement with the actual acquired data. It is noteworthy, that the intermediate denoised estimate of the diffusion model, calculated through Tweedie's Formula, needs to be transferred to the full FOV prior to the data consistency step. For this purpose, the periphery of the reconstruction used as an initialization—that is, the temporal average image—is attached again. Subsequently, DPS data consistency operations can be performed. For additional details on training and inference please refer to the Supporting Information.

### 2.4 Evaluation

To evaluate the proposed reconstruction method, free‐breathing and breath‐hold data from the remaining eight healthy volunteers and five patients were used.

Iterative reconstructions regularized by temporal total variation (TTV) [37, 42], low rank plus sparse (LRS) [43] as well as a 2D diffusion model served as baseline methods (see Supporting Information for details). Data were evaluated by quantitative image metrics and an expert reader study.

#### 2.4.1 Calculation of Quantitative Image Metrics

The following reference‐based scalar image metrics were assessed using the skimage.metrics library [44]: Structural similarity index measurement (SSIM), Peak signal to noise ratio (PSNR), and normalized root mean square error (NRMSE). Additional scalar metrics, which have recently been suggested for improved correlations with expert votes [45, 46] are provided as Supporting Information.

As this part of the evaluation requires a “gold standard” reference, segmented spiral cine acquisitions were assembled from breath‐held acquisitions, comprising a total of 104 equidistant spiral arms per frame. This dataset consisted of series from 70 slices from 6 healthy volunteers. Two healthy volunteers from the test dataset were discarded due to blurring in the segmented spiral cine, which would have corrupted the analysis.

Retrospectively undersampled “real‐time” frames were then simulated by applying Cartesian real‐time undersampling masks (as obtained by the GROG procedure, each containing a total of 13 equidistant spiral arms) onto the k‐space obtained by applying GROG on the data from several cardiac cycles (104 spiral arms). Subsequently, these simulated real‐time data were subjected to the proposed as well as to alternative reconstruction methods. Moreover, a reduced sampling mask with only seven spiral arms, for example, by leaving out every second arm of aforementioned real‐time masks prior to GROG, was retrospectively applied to the same data to assess the potential for further acceleration. Retrospectively undersampled data simulate temporal resolutions of 48 ms (13 spiral arms) and 26 ms (7 spiral arms).

All segmented cine and corresponding reconstructed images were cropped to image sizes of 120px × 120px, manually centered on the myocardium and rescaled to a data range of [0,1], before subjecting them to the comparison. Image metrics were assessed using a Friedman test as well as pair‐wise Wilcoxon signed‐rank tests with Bonferroni correction.

#### 2.4.2 Expert Reader Study

Image quality assessment was performed for real‐time, free‐breathing acquisitions by two expert readers (2–8 years of experience in cardiac imaging) in a blinded and fully randomized fashion. For the real‐time data from 8 healthy volunteers and 5 patients, a total of 40 temporal frames were reconstructed by applying the video diffusion model twice in two independent steps, that is, once to the first 20 frames of the series and then again to the second 20 frames (matrix size 20 × 304px × 304px each). Since the total duration of cardiac cycles for all participants were below 25 frames, the 40 frames at least covered one full cardiac cycle for each specimen.

These images were compared to reconstructions of the same data using the baseline methods (LRS, TTV, 2D diffusion) as well as to ECG‐gated Cartesian cine in breath‐hold.

Ratings were performed for the complete SAX stacks using an ordinal five‐point rating scale for the following items: Sharpness of the myocardial contours (5: excellent, 4: good, 3: moderate, 2: fair, 1: poor/non‐diagnostic), noise and undersampling artifacts (5: none, 4: very few, 3: moderate, 2: substantial, 1: non‐diagnostic).

Friedman tests as well as pair‐wise Wilcoxon signed‐rank tests with Bonferroni correction were performed on the averaged ratings of both expert readers.

## 3 Results

**FIGURE 2:**
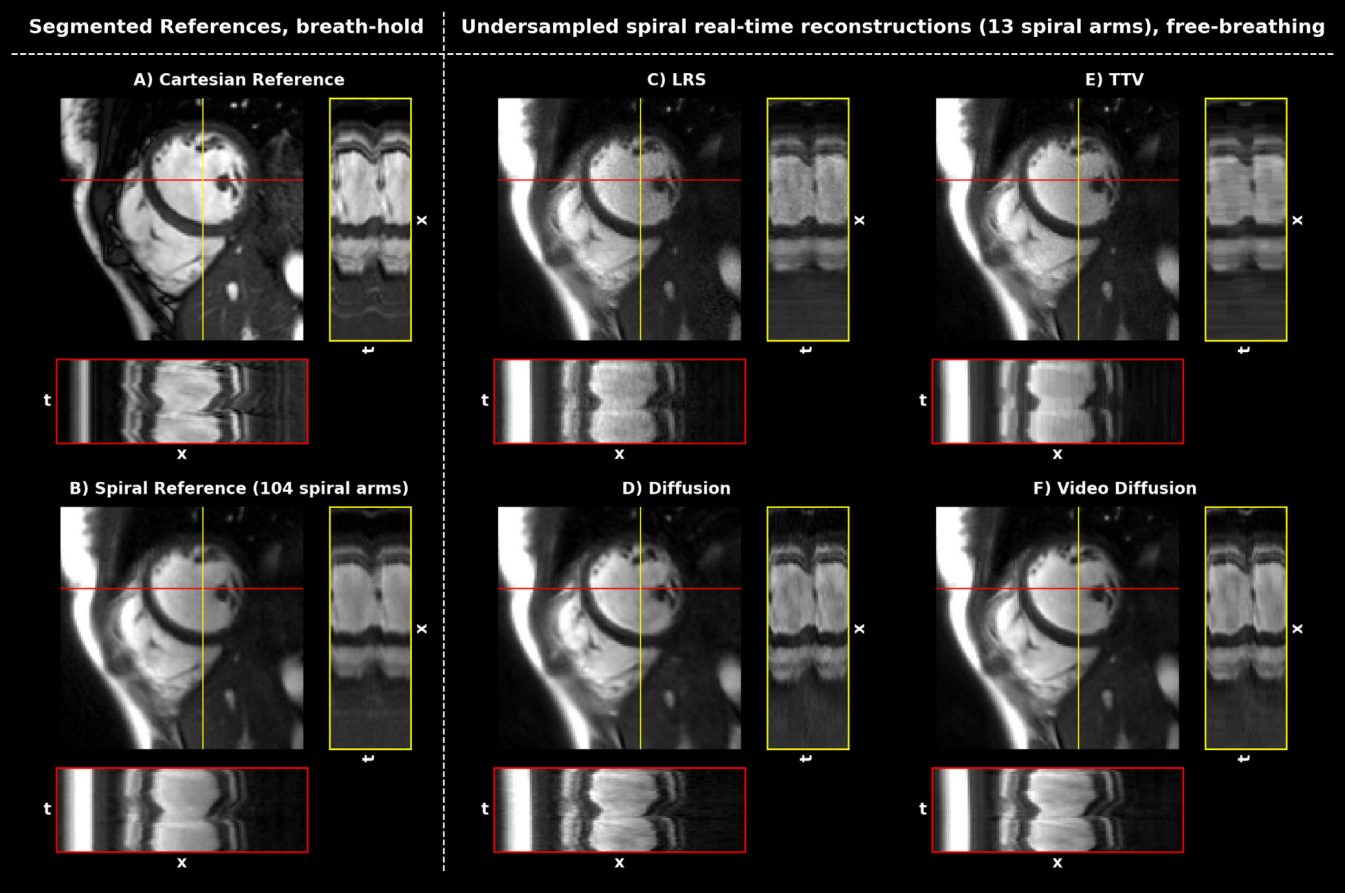
Overview of segmented breath‐held acquisitions (A) Cartesian, (B) spiral and (C–F) real‐time, free‐breathing acquisitions of a healthy subject with stable heartbeat reconstructed with the indicated reconstruction method. x–t plots depict cardiac motion in the direction of the shown vertical and horizontal lines. Since (A), (B), and (C–F) were acquired in separate measurements, slight variations in the depicted morphology can occur.

**FIGURE 3:**
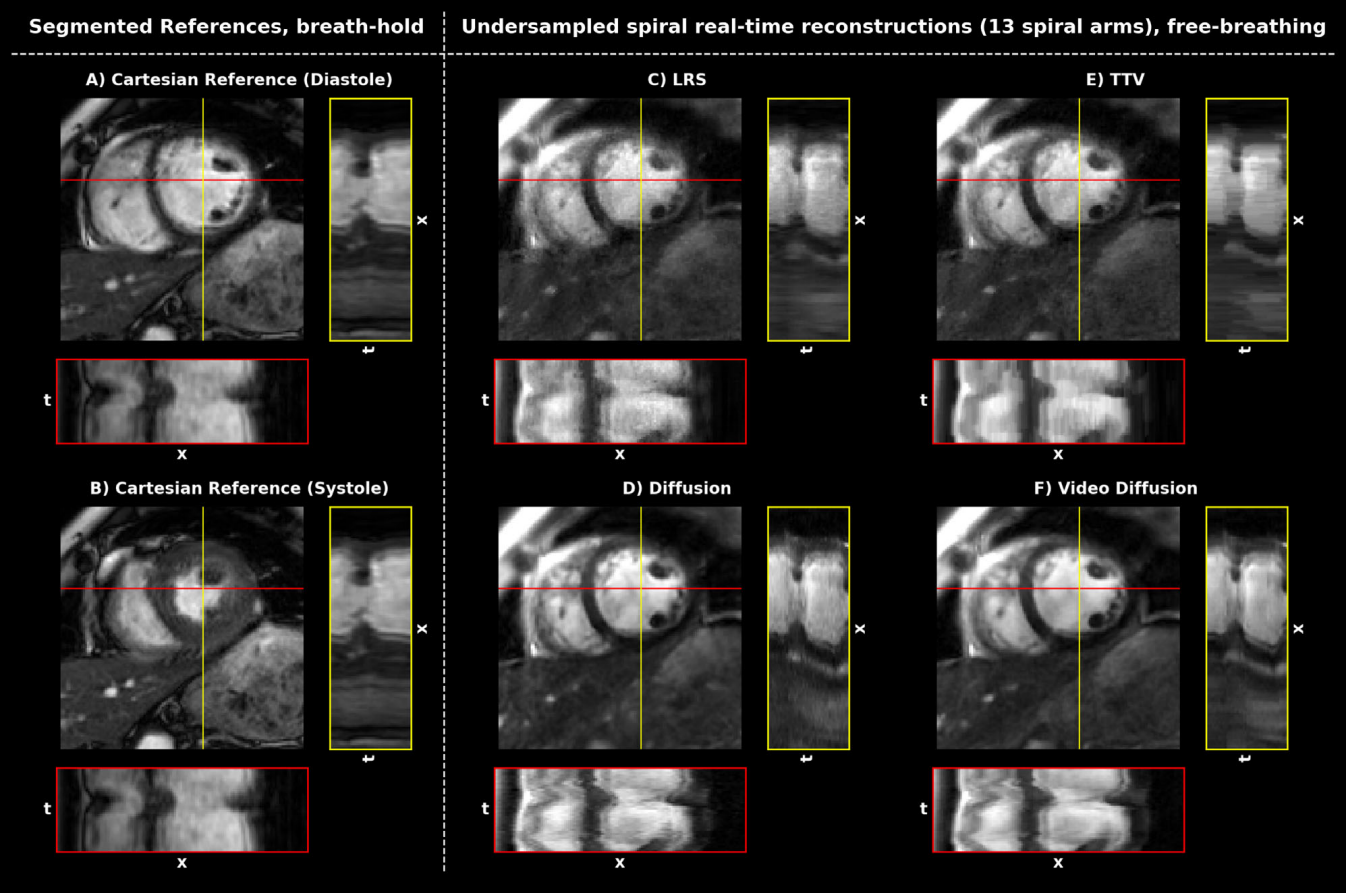
Overview of segmented breath‐held acquisitions (A) and (B) Cartesian and (C–F) real‐time, free‐breathing acquisitions of an arrhythmic patient reconstructed with the indicated reconstruction method. x–t plots depict cardiac motion along vertical and horizontal lines. Arrhythmia leads to severe blurring in the Cartesian reference, as seen in the x–t plots. Real‐time acquisitions are able to resolve cardiac motion accurately, with the video diffusion model offering sharp dynamics at reduced noise.

Figures 2 and 3 depict an overview of the results from acquisitions in breath‐hold and free‐breathing using the ECG‐gated Cartesian reference and the proposed spiral acquisition scheme for a healthy subject as well as a patient suffering from arrhythmia.

Reconstructions were cropped to the region of interest manually, centered on the myocardium and depicting approximately the same field of views. Since (A), (B), and (C–F) stem from consecutive measurements, however, slight differences of displayed morphologies occur.

Figure 2A,B shows segmented acquisitions in breath‐hold, while (A) was based on ECG‐gating and (B) on retrospective DC‐gating.

In Figure 3, the DC‐gated spiral reference is missing, since arrhythmia and failure to hold breath prevent sensible data binning. This is also reflected by temporally blurred depictions in the Cartesian case.

Figures 2C–F and 3C–F show reconstruction results from prospectively undersampled spiral‐real time frames acquired in free‐breathing and reconstructed with the indicated reconstruction method. For the healthy subject in Figure 2 all methods provide reconstructions with high visual image quality. The Cartesian reference appears somewhat sharper and shows a slightly different contrast compared to the spiral measurements, which can be explained by off‐resonance and trajectory misalignment effects, differences in the echo time and proprietary filtering algorithms routinely applied within clinical DICOM images. Slight temporal blurring can be seen in the x–t plots of the segmented spiral reconstruction in comparison to the real‐time, free‐breathing reconstructions. LRS, TTV, and 2D diffusion appear noisier than the video diffusion output, which also seems to perform best in depicting cardiac motion (see x–t plots). All reconstruction methods are essentially able to effectively remove undersampling artifacts in the region of interest. Figure 3 depicts a case where ECG‐gating fails, resulting in severe blurring of the clinical reference. Here, real‐time acquisitions offer a remedy and are able to accurately depict the dynamics. Reconstruction times were computed from 25 repeated reconstructions of 40 real‐time frames and were roughly ˜22 s per frame for the proposed method on a NVIDIA RTX A6000 (48GB), whereas the 2D diffusion model needed ˜36 s per frame. In comparison, LRS reconstructions took only ˜0.6 s per frame, while TTV reconstructions took ˜5 s per frame; however, running on CPU only.

Dynamic views for the data shown in Figures 2 and 3 can be found in Video S1 for the healthy subject and Video S2 for the patient.

Videos S3 and S4 depict full short axis stacks of the video diffusion reconstruction, depicting 40 real‐time frames acquired in free‐breathing. Minor edge effects appear at the periphery of the quadratic central crop regularized by the reconstruction procedure.

### 3.1 Calculation of Quantitative Image Metrics and Expert Reader Study

**FIGURE 4:**
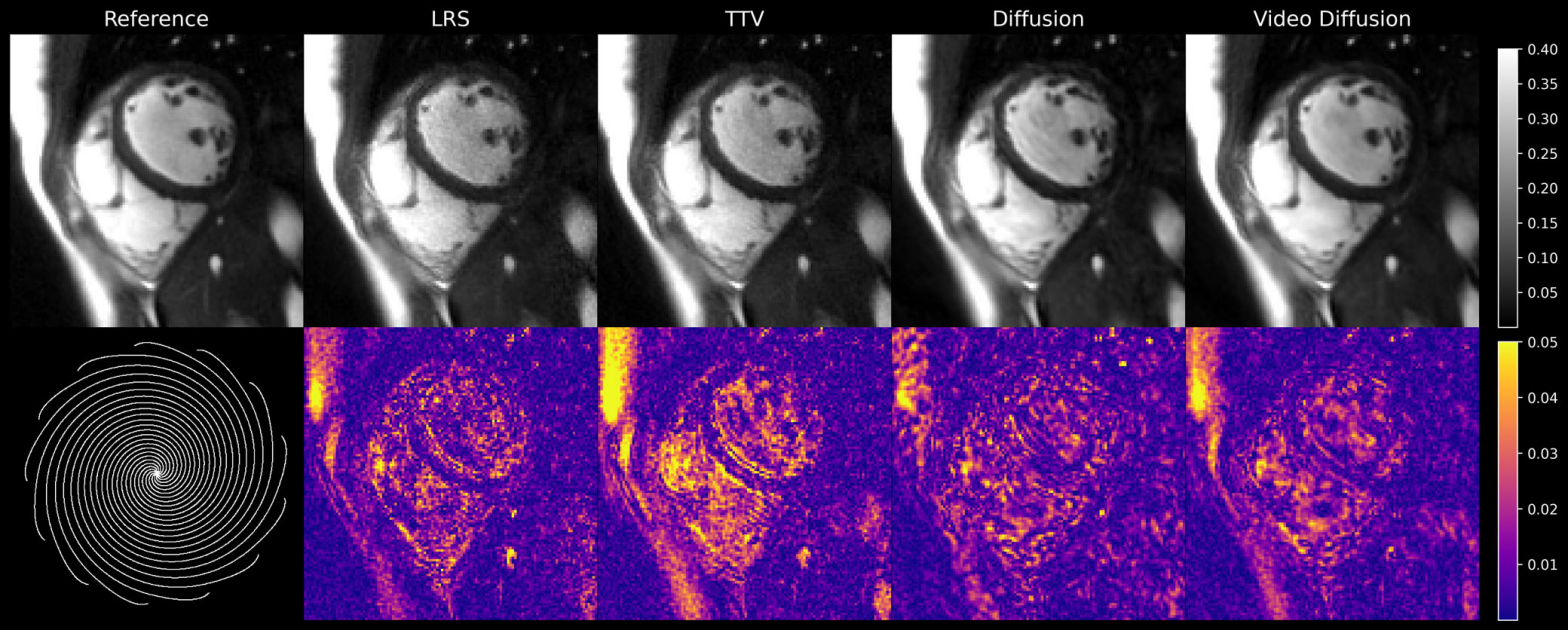
Comparison of different reconstruction techniques applied to retrospectively undersampled segmented spiral acquisitions (Reference). Error maps were computed between the spiral reference and respective reconstruction methods. The undersampling mask rotates approximately every 10 frames (also see Video S5). The video diffusion model performs best in the retrospective study as indicated by the quantitative metric scores listed in Table 1.

Table 1 and Table S1 list the results from the evaluation of quantitative metrics as described in Section 2. The video diffusion model performs best in all metric scores, indicating overall improved image quality in comparison. Figure 4 depicts an overview of a corresponding reconstruction from retrospectively undersampled breath‐held, segmented spiral acquisitions in combination with error maps.

**TABLE 1:** Results from the quantitative evaluation using scalar metrics and mean expert reader scores.

	Cartesian cine	LRS	TTV	2D Diffusion	Video diffusion
Image metrics					
SSIM (%)		93.0 ± 2.7	92.6 ± 2.5	91.4 ± 2.6	94.8 ± 2.0
NRMSE (%)		7.6 ± 2.0	7.9 ± 2.2	8.4 ± 2.0	6.8 ± 2.0
PSNR (dB)		36.0 ± 2.9	35.6 ± 2.9	35.1 ± 2.6	37.0 ± 3.0
Expert reader					
Sharpness	3.8 ± 0.9	4.1 ± 0.5	4.3 ± 0.5	4.1 ± 0.6	4.3 ± 0.6
Noise	4.2 ± 0.4	3.2 ± 0.4	3.5 ± 0.4	3.6 ± 0.5	4.2 ± 0.4
Undersampling artifacts	5.0 ± 0.1	3.9 ± 0.5	3.9 ± 0.6	3.8 ± 0.6	3.8 ± 0.6

*Note*: Results from the evaluation of quantitative metrics scores as well as the expert reader study as depicted in Section 2. The video diffusion model is able to outperform all baseline methods in the case of quantitative metrics. Expert reader results depict reduced noise levels at a preserved image sharpness and reduced under sampling artifacts for the proposed model.

Video S5 presents a dynamic view, showing all respective cine frames. LRS and TTV as well as 2D diffusion result in noisier reconstructions compared to the proposed model as indicated by larger deviations in the error maps. Temporal averaging in the case of the reference versus undersampled reconstructions leads to small structural differences near the border of the myocardium.

Quantitative metrics with the further accelerated sampling mask containing only seven spiral arms are listed in the Supporting Information (Table S2). Again, the proposed method performs best. Video S6 depicts a comparison between reconstruction results using the reduced sampling mask. These results suggest that further acceleration might be achievable without significantly compromising image quality. Friedman tests showed significant differences for each metric as well as each pairwise comparison for the proposed method (*p* < 0.05).

Additionally, mean results from the expert reader study are listed in Table 1 and with separate tables for both expert readers being shown in Table S3. Differences between reconstruction methods are small; however, they indicate that the proposed model reduces noise while preserving sharp reconstructions. This is supported by the statistics. Friedman tests resulted in significant differences for noise and the reduction of undersampling artifacts. For the proposed method, pair‐wise comparison resulted in significant differences for noise with respect to LRS and TTV (*p* < 0.05). In terms of undersampling artifacts, differences were significant between real‐time reconstructions and Cartesian cine (*p* < 0.05).

Temporal blurring leads to a decreased sharpness score in the case of the Cartesian cine.

## 4 Discussion

In this technical note, we proposed a real‐time technique for cardiac functional MRI based on spiral readouts and a video diffusion model for image reconstruction. In comparison with baseline methods, the presented method resulted in the best metric scores, indicating superior image quality.

Metrics determined for the accelerated sampling mask with only seven spiral arms reflected superiority of the diffusion model (see Supporting Information and Video S6), too, implying the possibility for further acceleration in prospectively undersampled acquisitions. Moreover, it underlines the adaptability of the diffusion models for different sampling masks. In general, scalar metrics are heavily dependent on the calculated region of interest, normalization, and quality of the reference and thus should not be exclusively consulted for the assessment of image quality.

Scores from the expert reader study indicated high diagnostic quality of real‐time images with reduced noise and low artifact power, while preserving sharpness for the proposed method.

Alternatively, real‐time cMR can also be facilitated using Cartesian golden angle sampling patterns as recently proposed by Vornehm et al. [12]. However, to reach the required spatial and temporal resolution, high acceleration factors of *R* ˜16 are needed. To overcome stark undersampling artifacts, the authors thus propose a novel variational network trained on segmented cine data. Other works have shown spiral‐in‐out trajectories as a means to provide contrasts closer to standard Cartesian acquisitions [47, 48]. Prolonged repetition times, however, can lead to off‐resonance induced blurring as well as banding artifacts in the region of interest. These effects can be mitigated using lower field strengths [48] or via correction methods using B0 maps [47].

The proposed method represents an extension to traditional 2D diffusion models, for example, as recently proposed for cardiac MRI [27]. By introducing a 2D + t network architecture, we improved temporal consistency in cardiac dynamics of real‐time reconstructions. As a temporal series is processed, reductions in reconstruction times were achieved in comparison to 2D processing. By focusing on the relevant region of interest for reconstruction, we complied with available memory restrictions, thus offering a more flexible approach to the reconstruction task.

### 4.1 Limitations

Long computation times of roughly ˜22 s per frame combined with the heavy computational burden of the video diffusion model prohibit immediate clinical translation. To be able to run the video diffusion model on our system, several interventions were needed, such as the introduction of gradient checkpointing and the application on a central crop only. In the course of the latter, we appended the periphery of a temporal average to the central crop to enforce data consistency in each iteration. In the case of heavy breathing motion, a temporal average might be insufficient for estimating the periphery. The addition of a SENSE‐like data consistency step also for the periphery could thus be beneficial.

Moreover, the network was trained exclusively on retrospectively segmented cine series acquired during breath‐hold. Therefore, the breathing motion in our free‐breathing real‐time acquisitions is not reflected by the diffusion model. We expect that this discrepancy may contribute to some blurring in the reconstruction output of free‐breathing data.

Furthermore, in this study the sampling pattern consisted of 13 equidistant spiral arms, which were rotated approximately every 0.5 s for free‐breathing acquisitions, mainly to avoid high frequent flickering of residual artifacts in the dynamic view. However, methods such as LRS or TTV potentially profit from higher rotation frequencies—for example, a golden angle for every other cardiac phase—possibly enabling an improved reconstruction performance here.

Finally, diffusion models are rather complex in nature and involve a large number of hyperparameters for training and inference. While this property enables high adaptability and fine parameter tuning for an optimized output, excessive optimizations are challenging due to the heavy computational burden, long training and inference times.

Finally, the sample size was small, and the statistical power of the evaluation would benefit from a larger cohort.

## 5 Conclusions

We proposed a video diffusion model to precisely model the data distribution of spiral MR scans in the myocardium, allowing for regularizing the reconstruction of accelerated spiral real‐time acquisitions for improved image quality and temporal consistency.

Even though computational burdens could be reduced by a training approach focusing on the region of interest only, they still present challenging hurdles, especially as reconstruction times are impractical for clinical routine with its current implementation.

## Funding

This work was supported by the German Ministry for Education and Research under Research Grant 05M20WKA and the Interdisciplinary Center for Clinical Research in Würzburg under Research Grant F‐437.

## Conflicts of Interest

Thorsten Alexander Bley discloses the following potential conflicts of interest; however, none of them are in the context of the publication mentioned: Financial research support of the department: Deutsche Forschungsgemeinschaft (DFG) and Siemens Healthineers: Personal consultant/speaker bureau: BioTel Research, Bracco, Chugai, GE, Guerbet, Roche, Novartis, and Siemens Healthineers. Viktor Hartung discloses the following potential conflicts of interest, however, none of them are in the context of the publication mentioned: speaker honoraria: Bracco.

## Supporting information


**Video S1:** Dynamic view with a temporal series of 40 frames comparing ECG‐gated Cartesian cine (A), retrospectively segmented spiral cine (B) both acquired in breath‐hold with real‐time free breathing reconstructions using the indicated methods: (C) LRS, (D) 2D Diffusion, (E) TTV and (F) the proposed video diffusion model for the healthy volunteer depicted in Figure 2.

**Video S2:** Dynamic view with a temporal series of 40 frames comparing ECG‐gated Cartesian cine (A) acquired in breath‐hold with real‐time free breathing reconstructions using the indicated methods: (C) LRS, (D) 2D Diffusion, (E) TTV and (F) the proposed video diffusion model for the patient depicted in Figure 3. The acquisition mask of free‐breathing real‐time acquisitions is shown in (B).

**Video S3:** Dynamic view of free‐breathing real‐time acquisitions reconstructed with the proposed video diffusion model depicting a temporal series of 40 frames covering the whole heart for the healthy volunteer shown in Figure 2.

**Video S4:** Dynamic view of free‐breathing real‐time acquisitions reconstructed with the proposed video diffusion model depicting a temporal series of 40 frames covering the whole heart for the patient shown in Figure 3.

**Video S5:** Dynamic view comparing reconstruction results with the indicated methods applied to retrospectively undersampled data using the acquisition mask of our free‐breathing real‐time acquisitions. Error maps depict deviations from the spiral reference.

**Video S6:** Dynamic view comparing reconstruction results with the indicated methods applied to retrospectively undersampled data using a reduced acquisition mask with 7 spiral arms only. Error maps depict deviations from the spiral reference.

**Data S1:** Supporting Information.

## Data Availability

Research data are not shared.
