# Supplementary material for: A Spatio‐Temporal Diffusion Model for Cardiac Real‐Time Imaging
Source: Magn Reson Med. 2026 Feb 18;95(6):3574–83. doi: 10.1002/mrm.70303 (PMC13049260; doi:10.1002/mrm.70303)
Supplement: Supplementary file 7 — Data S1: Supporting Information. [file MRM-95-3574-s001.docx]

# Supplementary Information

# Training details of the spatio-temporal diffusion model

Video diffusion models were proposed as the extension of the 2D spatial diffusion approach, e.g. for generating temporally coherent dynamic image series. Originally introduced by Ho et al.^1^, generative tasks such as unconditional video generation, text conditioned video generation, video prediction & extension were investigated. The authors employed a 2D+t-Unet architecture factorized over time and space. While convolutional layers work on spatial dimensions only and treat the time axis as a batch axis, additional temporal attention layers act between sequential images to create coherence between frames. In our work we use an openly available implementation adapting the video diffusion model network architecture^1,2^.

We refer to ^3–6^ for a detailed description of generative modeling using stochastic differential equations as well as conditioned sampling via Diffusion Posterior sampling (DPS) and will only give a brief description of our parameters used for training.

For the latter, the minimization given by $\underset{\Theta}{\arg\min} E_{t, x\left( 0 \right),x(t)|x\left( 0 \right)}[{||\sigma\left( t \right)s_{\Theta}\left( x\left( t \right),\sigma\left( t \right) \right)+z||}_{2}^{2}]$ was realized using denoising score matching. $s_{\Theta}\left( x\left( t \right),\sigma\left( t \right) \right)$ depicts the 2D+t video diffusion model used to estimate the noise $\sigma(t)$ in each image of the time series $x\in\mathbb{C}^{18 x 304 x 304}$. The noise scale represented a geometric sequence $\sigma\left( t \right)=\sigma_{min}\left( \frac{\sigma_{max}}{\sigma_{min}} \right)^{t}$ and for the training, a random timestep $t \in[0,1]$ was chosen with uniform probability to perturb the training data. Noisy image series are given by $x\left( t \right)=x_{0}+\sigma\left( t \right) z$, with $x_{0}$ resembling the unperturbed training data and z depicting standard Gaussian noise $\mathcal{N}\left( 0, I \right)$. In practice, a discretization into $N$ discrete timesteps of the continuous time interval was used.

The noise scale for training the network followed a geometric series with $\sigma_{min}=0.005$ and $\sigma_{max}=1$. A total of $N=500$ diffusion steps were trained using Adam optimizer with a batch size of 1 and a linear learning rate warm up, that reached a constant learning rate of $2*{10}^{-4}$ after 5000 steps with $\beta_{1}=0.9$ and $\beta_{2}=0.9$99 and an exponential moving average rate of 0.999. Modelling the score function involved discretizing the timesteps $t\in[0,1]$ into $N$ noise scales $t_{i}=\frac{i-1}{N-1}$ with $i\in[1,N]$. Training was performed for 2500 epochs on a NVIDIA RTX A6000 GPU with 48 GB memory.

**Diffusion Posterior Sampling (DPS)**

During inference, an aliasing-free complex temporal average $x_{mean}\in\mathbb{C}^{N_{fr} x 512 x 512}$, normalized with respect to its maximum magnitude value and repeated for $N_{fr}$ frames in the temporal dimension, was used for initialization. For reconstruction, a reduced number of $N=100$ noise scales were used with $\sigma_{min}=0.01$ and $\sigma_{max}=0.1$, which were chosen empirically. The sampling procedure thus began with $x_{init}= x_{mean}+0.1 z$. Data consistency was performed using the forward operator $A=M\mathcal{F}S_{N_{c}}$, where $\mathcal{F}$ is a fast 2D-Fourier-transform, $S_{N_{c}}$ are coil sensitivity maps and $M$ is the undersampling mask of the acquired multi-coil data $y\in\mathbb{C}^{N_{fr} x N_{c} x 512 x 512}$. Undersampled k-spaces were also normalized with respect to the maximum magnitude value of the temporal average image used for initialization of the reconstruction.

Inspired by Chung et al. ^6^, the scaling $\gamma$ of the data consistency for DPS sampling was set to $\gamma_{i}=5/\left| \left| y-A{\hat{x}'}_{0}\left( x_{i} \right) \right| \right|_{2}$, where $\hat{x}_{0}\left( x_{i} \right)=x_{i}+\sigma_{i}^{2}s_{\Theta}\left( x_{i},\sigma_{i} \right)$represents an intermediate denoised estimate at time $t=0$, calculated through Tweedie’s Formula^7^. As outlined in section 2.3 in the manuscript, the model was trained and applied on a central crop only, in order to maintain our memory restrictions. Thus, to perform DPS and match the dimensions of the acquired data $y$, the periphery of the temporal average was repeatedly attached to the intermediate denoised estimates, such that ${\hat{x}'}_{0}\left( x_{i} \right)=M_{outer}\odot x_{mean}+\mathrm{Pad}_{512}\left( \hat{x}_{0}\left( x_{i} \right) \right)$. $M_{outer}$ and $\mathrm{Pad}_{512}$ represent sampling masks for the periphery and zero-padding of the central region to spatial dimensions of 512px × 512px, e.g. the original dimensions of the initially gridded data using GROG.

An overview of the sampling procedure is depicted in the algorithm shown below as well as the schematic illustration by Figure 1 in the manuscript.

Our source code for training and inference will be publicly available upon publication <https://github.com/expRad?tab=repositories>.

**Algorithm 1: Physics-based image reconstruction exploiting a spatio-temporal diffusion model**

Require: $s_{\Theta}, N,\left\{ \sigma\right\}_{i=1}^{N}, \sigma_{0}=0, x_{mean}, y$

$x_{N}=x_{mean}+\sigma_{N}\mathcal{N}\left( 0, I \right)$ noise perturbed complex temporal average reconstruction

$x_{N}=center(x_{N},304)$ center crop on full sized image stack

For i=N:1 do:

$x_{i-1}=x_{i}+\left( \sigma_{i}^{2}-\sigma_{i-1}^{2} \right)s_{\Theta}\left( x_{i},\sigma_{i} \right)$ reverse diffusion sampling

If i!=1: $x_{i-1}=x_{i-1}+ \sqrt{\sigma_{i}^{2}-\sigma_{i-1}^{2}}\mathcal{N}(0,I)$

$\hat{x}_{0}\left( x_{i} \right)=x_{i}+\sigma_{i}^{2}s_{\Theta}\left( x_{i},\sigma_{i} \right)$ denoised estimate at $t=0$

${\hat{x}'}_{0}\left( x_{i} \right)=M_{outer}\odot x_{mean}+\mathrm{Pad}_{512}\left( \hat{x}_{0}\left( x_{i} \right) \right)$ combination of the denoised estimate with the periphery of the temporally averaged initialization

$x_{i-1}=x_{i-1}-\gamma_{i}\nabla_{x_{i}} \left| \left| y-A{\hat{x}'}_{0}\left( x_{i} \right) \right| \right|_{2}^{2}$ DPS data consistency step

End for

Return $x_{0}$

Caption: Reconstruction algorithm for dynamic real-time cMR acquisitions using a spatio-temporal diffusion model.

# Memory estimation working on full matrices

Memory limitations are the primary motivation for our approach of working on a reduced matrix size. Complementary, we here provide an estimate for the memory consumption on full resolution datasets.

The network architecture consists of convolutional layers as well as attention blocks and is complex in nature. We therefore refrain from a theoretical derivation of extrapolated memory consumption. However, since the model is flexible in terms of temporal and spatial dimensions, we record memory usage during inference for varying temporal and spatial dimensions. Please note that inference is also requiring tracking of gradients due to the diffusion posterior sampling step, similar to training. We thus restrict the evaluation to the inference procedure only.

Results from this evaluation are shown in Figure S2 depicting linear fits $y=mx+b$ applied to data points with fixed spatial dimensions and varying temporal dimensions and quadratic fits $y=ax^{2}+bx+c$ applied to points with fixed temporal dimension and varying spatial dimensions. Extrapolated memory demands from the fits for a spatial dimension of 512px×512px are 187-197GB for 40 temporal frames and 372-438GB for 80 temporal frames.

# Baseline methods used in the evaluation of quantitative metrics

To evaluate reconstruction performance, the following baseline methods were used for comparison with the image quality obtained using the proposed video diffusion model, as outlined in Section 2 of the manuscript. Keep in mind that prior to reconstruction, undersampled spiral data were transferred to Cartesian grids using Grappa Operator Gridding (GROG)^8^ with spatial dimensions of 512px×512px.

*Temporal total variation (TTV):*

Model based temporal total variation^9^ reconstructions are performed on a full 2D+t image series using BART^10^. The k-spaces of retrospectively undersampled acquisitions were normalized with respect to the maximum value of the whole image stack of coil-combined undersampled images. The minimization problem was solved using 50 ADMM iterations with a regularization parameter of $\lambda=0.08$. The regularization parameter was chosen by optimizing SSIM based on retrospectively undersampled and reconstructed mean data from 8 central short axis slices with 24 frames each (192 individual images total) from a single subject of test dataset.

*Low Rank plus Sparse (LRS):*

On the same 2D+t series, low rank plus sparse (L+S) reconstructions were performed, enforcing sparsity by thresholding the singular values of a Casorati matrix as well as thresholding of temporal frequencies^11^. Regularization parameters were set to $\lambda_{L}=0.01$ and $\lambda_{S}=0.06$ and a total of 60 iterations were performed. Data normalization and determination of regularization parameters were performed analogously to the TTV case.

*2D Diffusion model*:

The utilized video diffusion network architecture was converted into a 2D diffusion model by setting the training input dimensions to 1 x 304px×304px. Thereby, only one randomly selected frame of the spiral cine training set was used per training step here.
For Diffusion Posterior Sampling the data scaling factor was reduced to $\gamma_{i}=1/\left| \left| y-A{\hat{x}'}_{0} \right| \right|_{2}$. Otherwise, identical training and reconstruction parameters were used as in the case for the 2D+t video diffusion model.

# Calculation of alternative quantitative metrics

Even though SSIM, PSNR and NRMSE are well established reference-based metrics, the following metrics have partly shown improved correlations with expert votes^12^: Deep Image Structure and Texture Similarity (DISTS), Haar wavelet-based Perceptual Similarity Index (Haar PSI) and Visual Saliency-based Index (VSI). Therefore, the latter were additionally calculated using the openly available pytorch image quality library^13^. Haar PSI and VSI range from 0 (worst) – 1 (best), while DISTS is unbounded 0 (best) - ∞ (worst).

In our case, however, these metrics aligned well with the standard metrics, also being best for the proposed video diffusion model (see Table S1 and Table S2).

**References of the supplementary information**

1. Ho J, Jain A, Abbeel P. Denoising Diffusion Probabilistic Models. In: Larochelle H, Ranzato M, Hadsell R, Balcan MF, Lin H, eds. *Advances in Neural Information Processing Systems*. Vol 33. Curran Associates, Inc.; 2020:6840-6851. https://proceedings.neurips.cc/paper_files/paper/2020/file/4c5bcfec8584af0d967f1ab10179ca4b-Paper.pdf.

2. Wang P. video-diffusion-pytorch. October 2023. https://github.com/lucidrains/video-diffusion-pytorch.

3. Song Y, Sohl-Dickstein J, Kingma DP, Kumar A, Ermon S, Poole B. Score-Based Generative Modeling through Stochastic Differential Equations. In: *International Conference on Learning Representations*. ; 2021. https://openreview.net/forum?id=PxTIG12RRHS.

4. Chung H, Ye JC. Score-based diffusion models for accelerated MRI. *Medical Image Analysis*. 2022;80:102479. doi:10.1016/j.media.2022.102479

5. Chung H, Sim B, Ye JC. Come-Closer-Diffuse-Faster: Accelerating Conditional Diffusion Models for Inverse Problems Through Stochastic Contraction. In: *Proceedings of the IEEE/CVF Conference on Computer Vision and Pattern Recognition (CVPR)*. ; 2022:12413-12422.

6. Chung H, Kim J, Mccann MT, Klasky ML, Ye JC. Diffusion Posterior Sampling for General Noisy Inverse Problems. In: *The Eleventh International Conference on Learning Representations*. ; 2023. https://openreview.net/forum?id=OnD9zGAGT0k.

7. Efron B. Tweedie’s Formula and Selection Bias. *Journal of the American Statistical Association*. 2011;106(496):1602-1614. doi:10.1198/jasa.2011.tm11181

8. Seiberlich N, Breuer FA, Blaimer M, Barkauskas K, Jakob PM, Griswold MA. Non‐Cartesian data reconstruction using GRAPPA operator gridding (GROG). *Magnetic Resonance in Med*. 2007;58(6):1257-1265. doi:10.1002/mrm.21435

9. Block KT, Uecker M, Frahm J. Undersampled radial MRI with multiple coils. Iterative image reconstruction using a total variation constraint. *Magnetic Resonance in Med*. 2007;57(6):1086-1098. doi:10.1002/mrm.21236

10. Blumenthal M, Holme C, Roeloffs V, et al. mrirecon/bart: version 0.8.00. September 2022. doi:10.5281/ZENODO.7110562

11. Otazo R, Candès E, Sodickson DK. Low-rank plus sparse matrix decomposition for accelerated dynamic MRI with separation of background and dynamic components: L+S Reconstruction. *Magn Reson Med*. 2015;73(3):1125-1136. doi:10.1002/mrm.25240

12. Kastryulin S, Zakirov J, Pezzotti N, Dylov DV. Image Quality Assessment for Magnetic Resonance Imaging. *IEEE Access*. 2023;11:14154-14168. doi:10.1109/ACCESS.2023.3243466

13. Kastryulin S, Zakirov D, Prokopenko D. PyTorch Image Quality: Metrics and Measure for Image Quality Assessment. 2019. https://github.com/photosynthesis-team/piq.


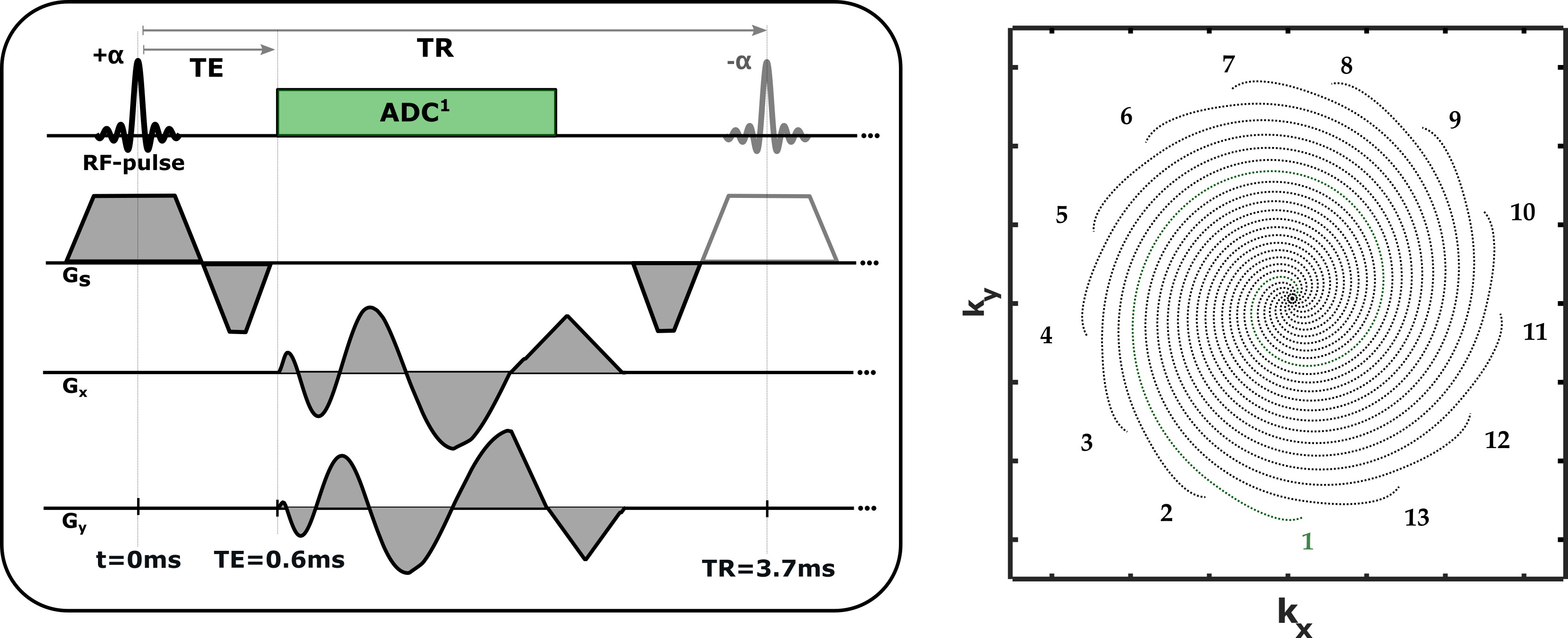


Figure S1: Schematic pulse sequence design for the acquisition of accelerated spiral cardiac real-time images. Undersampled images consist of 13 equidistant spiral arms, as depicted on the right.


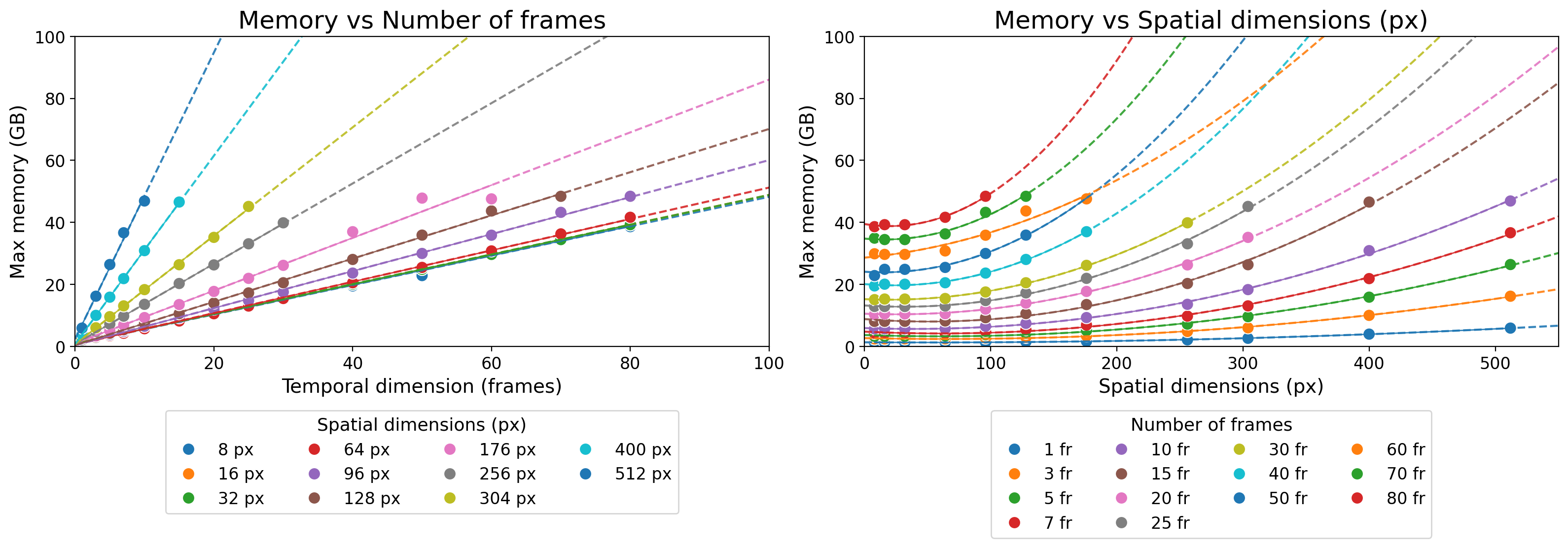


Figure S2: Estimate for the memory demands when working with different temporal and spatial dimensions during inference. Linear fits $y=mx+b$ were applied to curves with fixed spatial dimensions and varying temporal dimensions. Quadratic fits $y=ax^{2}+bx+c$ were applied to points with fixed temporal dimension and varying spatial dimensions. From the fits, estimates for the memory consumption at full spatial resolution 512px x 512px and 40 as well 80 frames temporal frames were extrapolated.

**Table S1: Results from the quantitative evaluation of alternative scalar metrics**

| Image metrics | LRS | TTV | 2D Diffusion | Video Diffusion |
| --- | --- | --- | --- | --- |
| DISTS [%] | 12.6±2.1 | 12.6±2.0 | 13.5±2.0 | **9.1±1.7** |
| Haar-PSI [%] | 90.4±4.4 | 90.3±4.4 | 86.9±4.8 | **91.6±4.2** |
| VSI [%] | 99.0±0.5 | 99.0±0.5 | 98.8±0.5 | **99.3±0.4** |

Table S1: Results from the evaluation of alternative quantitative metrics scores. These metrics aligned well with the standard metrics, also being best for the proposed video diffusion model.

**Table S2: Results from the quantitative evaluation using scalar metrics adopting a higher undersampling**

| Image metrics | LRS | TTV | 2D Diffusion | Video Diffusion |
| --- | --- | --- | --- | --- |
| SSIM [%] | 80.8±4.1 | 81.3±4.0 | 86.1±3.6 | **92.5±2.2** |
| NRMSE [%] | 18.3±4.8 | 15.8±3.8 | 11.8±3.2 | **8.2±1.6** |
| PSNR [dB] | 28.4±2.5 | 29.6±2.3 | 32.1±2.4 | **35.1±2.2** |
| DISTS [%] | 23.8±2.2 | 22.9±2.1 | 17.5±2.0 | **11.3±2.0** |
| Haar-PSI [%] | 73.9±6.2 | 75.2±5.6 | 78.6±5.5 | **87.9±4.1** |
| VSI [%] | 97.8±1.0 | 97.9±0.9 | 98.2±0.7 | **99.1±0.4** |

Table S2: Quantitative scores as depicted in Section 2 for higher undersampling (7 spiral arms per frame, see Video S6). The video diffusion model outperforms all baseline methods, indicating the possibility for further acceleration in prospectively undersampled acquisitions.

**Table S3: Results from expert reader study listing results for each expert separately**

| Expert reader #1 | Cartesian cine | LRS | TTV | 2D Diffusion | Video Diffusion |
| --- | --- | --- | --- | --- | --- |
| Sharpness | 3.8±1.0 | 4.7±0.5 | **4.9**±0.3 | 4.2±0.7 | **4.9**±0.4 |
| Noise | **4.6**±0.5 | 2.9±0.3 | 3.4±0.6 | 3.5±0.8 | 4.5±0.5 |
| Undersampling artifacts | **5.0**±0 | 4.2±0.9 | 4.5±0.8 | 4.2±0.7 | 4.2±0.7 |
| Expert reader #2 | Cartesian cine | LRS | TTV | 2D Diffusion | Video Diffusion |
| Sharpness | 3.8±0.9 | 3.5±0.9 | 3.7±0.8 | **4.0**±0.7 | 3.8±1.0 |
| Noise | **3.9**±0.7 | 3.5±0.6 | 3.6±0.5 | 3.7±0.5 | **3.9**±0.9 |
| Undersampling artifacts | **4.9**±0.3 | 3.5±0.6 | 3.2±0.6 | 3.4±0.7 | 3.4±0.9 |

Table S3: Separate results from the expert reader study as outlined in Section 2 in the manuscript.
